# Growth factor restriction impedes progression of wound healing following cataract surgery: identification of VEGF as a putative therapeutic target

**DOI:** 10.1038/srep24453

**Published:** 2016-04-14

**Authors:** Julie A. Eldred, Matthew McDonald, Helen S. Wilkes, David J. Spalton, I. Michael Wormstone

**Affiliations:** 1 School of Biological Sciences, University of East Anglia, Norwich NR4 7TJ, UK; 2 King Edward VII Hospital, London, UK

## Abstract

Secondary visual loss occurs in millions of patients due to a wound-healing response, known as posterior capsule opacification (PCO), following cataract surgery. An intraocular lens (IOL) is implanted into residual lens tissue, known as the capsular bag, following cataract removal. Standard IOLs allow the anterior and posterior capsules to become physically connected. This places pressure on the IOL and improves contact with the underlying posterior capsule. New open bag IOL designs separate the anterior capsule and posterior capsules and further reduce PCO incidence. It is hypothesised that this results from reduced cytokine availability due to greater irrigation of the bag. We therefore explored the role of growth factor restriction on PCO using human lens cell and tissue culture models. We demonstrate that cytokine dilution, by increasing medium volume, significantly reduced cell coverage in both closed and open capsular bag models. This coincided with reduced cell density and myofibroblast formation. A screen of 27 cytokines identified nine candidates whose expression profile correlated with growth. In particular, VEGF was found to regulate cell survival, growth and myofibroblast formation. VEGF provides a therapeutic target to further manage PCO development and will yield best results when used in conjunction with open bag IOL designs.

Cataract is the leading cause of blindness worldwide^1^. It is estimated that by the year 2020 that 32 million people will require cataract removal surgery^2^. While cataract surgery initially yields a good restoration of vision, secondary visual loss results following a wound-healing response within the remaining lens tissue; this leads to cells encroaching within the visual axis, deforming the underlying collagenous posterior capsule and depositing additional matrix components. These changes cause light scatter and consequently visual deterioration, which is known as posterior capsule opacification (PCO), “after-cataract” or “secondary cataract”. PCO is treated by ablation of light-scattering material from within the visual axis using laser surgery. With an ever-increasing life expectancy, cataract and PCO will become an even greater problem, both in terms of patient well-being^3^ and economic burden^4^. Therefore, further advances in our understanding of this condition is essential to develop better management in the future.

An important part of most cataract surgeries is to implant an artificial intraocular lens (IOL), which sits within the remaining lens capsules, which are commonly referred to as the capsular bag. The capsular bag hosts the IOL to maintain stability and position, which allows best restoration of visual quality. Design and material modifications to IOLs in the past 20 years have significantly improved and reduced the time to PCO presentation^5^. However, the increase in cataract surgeries and our increasing aging population is now countering this reduction. 1.82 million cataract operations are performed in the USA annually, costing Medicare $3.4 billion each year^4^, and making cataract surgery the most common surgical procedure performed by healthcare providers. Furthermore, recent studies are demonstrating that the rates of clinically relevant PCO development at 10 years post cataract surgery are rising^6^. Although cataract is regarded as an aging disorder, the age of cataract removal is decreasing, perhaps as a consequence of populations working further into old age and therefore a greater necessity for good vision. Certainly in many countries the age at which cataract surgery is undertaken has decreased^6^. PCO therefore continues to be a considerable problem associated with cataract patient care.

IOL designs currently incorporate a square-edge motif on the posterior side of the IOL optic and rely on a closed capsular bag IOL arrangement. As the capsular bag “shrink wraps” the IOL subsequent to implantation the square-edge works as a physical barrier pressing into the posterior capsule preventing invading lens epithelial cells progressing on to the posterior capsule^7^. This effectively delays the time for PCO to present. However, eventually this physical barrier is breached and the lens epithelial cells rapidly proceed onto the posterior capsule. More recent studies on innovative IOL devices are demonstrating a way to further impact on posterior cell cover compared with classical closed capsular bag IOL systems. These open capsular bag designs keep the posterior and anterior capsules separated following IOL implantation^8^,^9^,^10^. This allows aqueous humour into the capsular bag space and this flow of aqueous humour is thought to help wash-out and reduce growth factor levels contained within the capsular bag that are upregulated as a response to cataract surgery.

Gaining a greater understanding of the processes that govern PCO progression and the benefits of IOL design are essential if we are to produce effective, sustainable and affordable therapies for eliminating PCO. The purpose of this study was to use human cell and tissue models to test the hypothesis that growth factor dilution (predicted with open bag IOLs) could reduce PCO and to better understand which growth factors and cytokines play key roles in PCO related events. Our study found that a general reduction in growth factor availability significantly impeded cell invasion on the posterior capsule and myofibroblast expression. These findings support the idea of open bag IOLs for use in cataract surgery. Moreover, we determined that a number of cytokines could influence growth characteristics of human lens cells and in particular it was found that VEGF plays a key role in growth and transdifferentiation. Consequently, inhibition of VEGF/VEGFR signalling is a logical target for future strategies to prevent PCO development in addition to improved IOL design.

## Results

### Increasing the volume of bathing media results in a decrease in cell cover on the posterior capsule of human capsular bags

**Figure 1:**
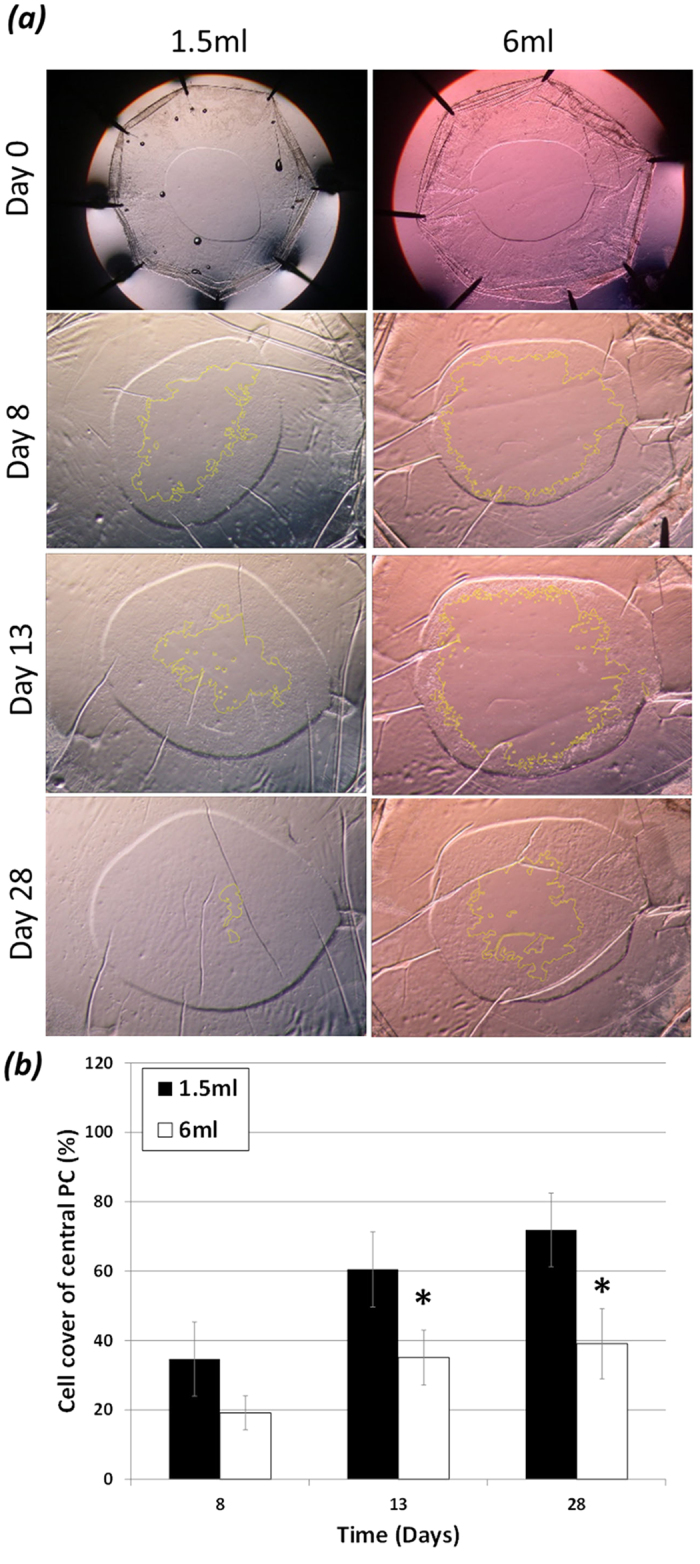
Posterior capsule cell cover: (**a,b**) Comparison of 1.5 ml and 6 ml SF EMEM treated match-paired capsular bags cultured over a 28-day period. (**a**) Phase micrographs at day 0 showing no cell cover on the central posterior capsule viewed via the anterior capsule rhexis. (**a,b**) Cell progression has advanced onto the central posterior capsule region at day 8 of culture in both 1.5 ml and 6 ml cultures, this cell cover is not significantly different. (**a,b**) At Day 13 significantly greater cell cover is observed on the central posterior capsule in 1.5 ml cultures compared to 6 ml counter. (**a,b**) Day 28 (end-point) of culture almost complete cell cover of the central posterior capsule observed in 1.5 ml capsular bags compared with a significant reduction in cell cover in 6 ml counterparts. The data are expressed as Mean ± SEM (*n* = 10). *Indicates significant difference between the 1.5 ml and 6 ml groups (P ≤ 0.05, Students t test). Average age of donor was 72.8 ± 2.7 years.

The coverage of cells on the anterior capsule following surgery was observed and found to vary between donors, but was similar with respect to capsular bags from the same donor. With culture, there was no discernible difference in the point in time at which cells passed the rhexis edge on to the previously cell-free central posterior capsule in capsular bags cultured in 1.5 ml or 6 ml SF (serum-free) EMEM (4.2 ± 0.9 and 3.9 ± 1.1 days respectively). A notable difference in cell cover was observed at day 8 between the 1.5 ml and 6 ml cultures. 7 out of 10 capsular bag pairs had greater cover on the posterior capsule within the rhexis region at this time point in the 1.5 ml compared to the 6 ml cultures, and 2 out of the 10 capsular bag pairs had no cell cover in the rhexis region in either the 1.5 ml or the 6 ml cultures. However, overall no significant difference was observed (Fig. 1). By culture day 13 the difference in cell cover between the match-pairs was significant, such that there was 60.5 ± 10.8% cover in the 1.5 mls and a 35.1 ± 7.9% cover in the 6 ml capsular bag cultures (Fig. 1). At day 28 (end-point) the 1.5 ml capsular bags still had significantly greater cell cover on the central posterior capsule region compared to 6 ml counterparts, such that coverage was 71.8 ± 10.6% and 39.1 ± 10.1% respectively (Fig. 1).

### Cell density is decreased with increasing media volume in human capsular bags

**Figure 2:**
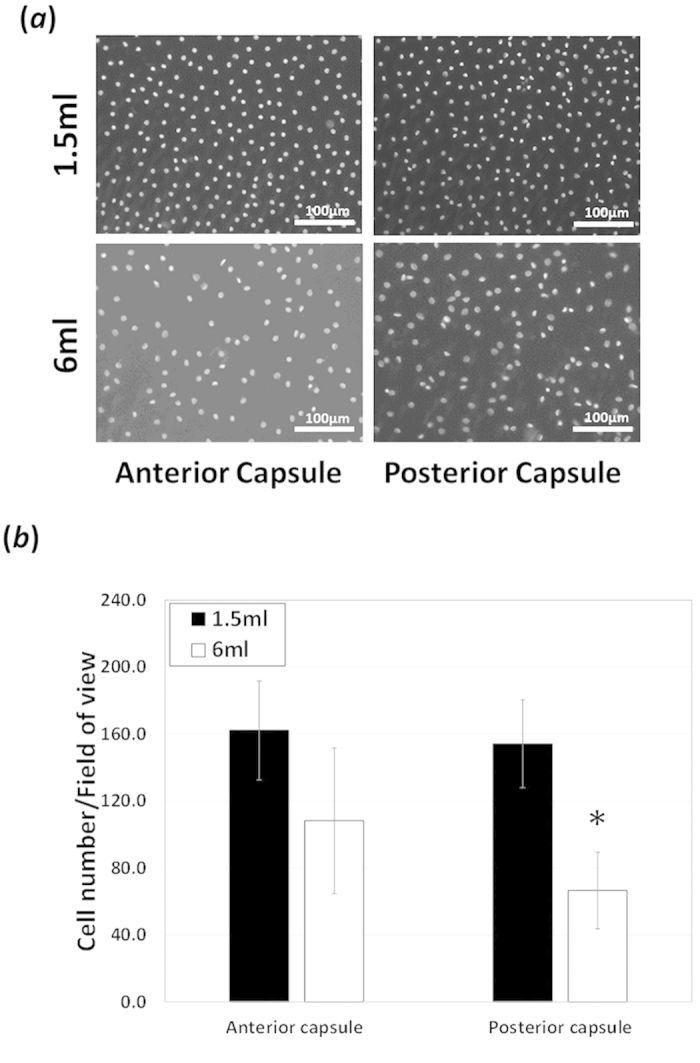
Cell density decreases with media dilution: (**a,b**) Comparison of cell population numbers in 1.5 ml and 6 ml SF EMEM treated match-paired capsular bags cultured for 28-days. (**a**) Epifluorescence micrographs at day28 showing a marked decrease in cell numbers on the anterior capsule in 6 ml culture compared to 1.5 ml counter-parts. Cell population numbers were determined by chromatin staining with DAPI (4′,6-diamidino-2-phenylindole). This decrease in cell number was not significantly different. (**a,b**) Epifluorescence micrographs at day 28 showing a significant decrease in cell numbers on the posterior capsule in 6 ml culture compared to 1.5 ml counter-parts. The data are expressed as Mean ± SEM (*n* = 8). *Indicates significant difference between the 1.5 ml and 6 ml groups (P ≤ 0.05, Students t test).

The cell density on the posterior capsule region of closed capsular bag cultures at end-point (day 28) was found to be significantly less in 6 ml SF EMEM cultures compared to 1.5 ml cultures (66.6 ± 22.9 and 154.3 ± 26.2 respectively, Fig. 2). Additionally, the number of cells in the anterior capsule region was markedly reduced in the 6 ml cultures compared to the 1.5 ml but this difference was not significant (Fig. 2).

### Epithelial Mesenchymal Transition (EMT) is decreased in cells on the posterior capsule with increased medium volume in human capsular bag cultures

**Figure 3:**
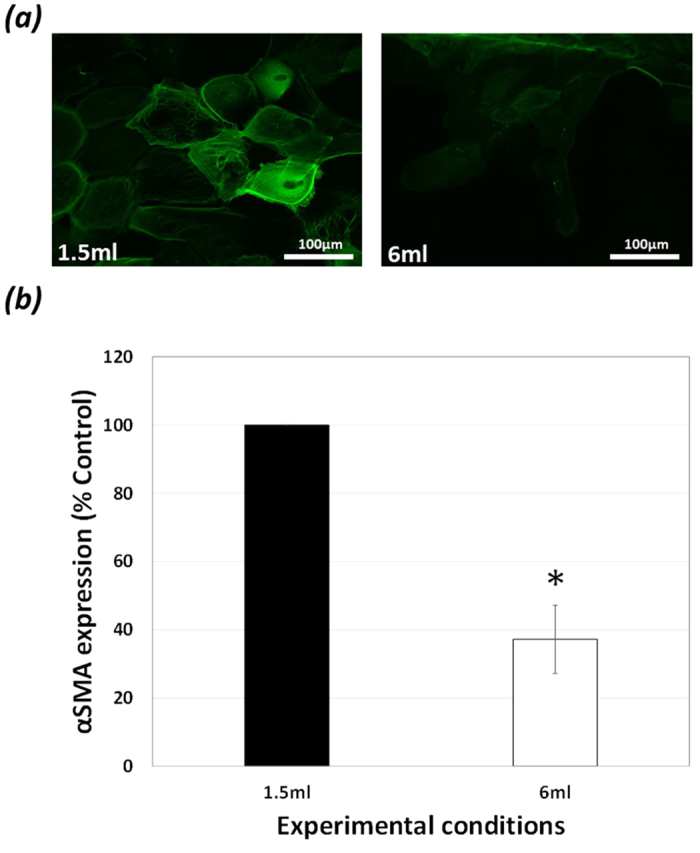
EMT decreases with media dilution: (**a,b**) Comparison of the expression of myofibroblastic cell marker αSMA in 1.5 ml and 6 ml SF EMEM treated match-paired capsular bags cultured for 28-days. (**a,b**) Epifluorescence micrographs at day 28 show a significant decrease in αSMA expression in cells on the central posterior capsule in 6 ml cultures compared to 1.5 ml counter-parts. The data are expressed as Mean ± SEM (*n* = 8). *Indicates significant difference between the 1.5 ml and 6 ml groups (P ≤ 0.05, Students t test). αSMA expression shown in green.

In order to evaluate EMT on the capsular bag, the well-established myofibroblast marker αSMA was determined^11^. At end-point (day 28) a significant reduction in αSMA expression was observed in cells growing on the posterior capsule within the capsulorhexis region of capsular bags cultured in 6 ml SF EMEM compared with levels observed in counterparts maintained in 1.5 ml SF EMEM, such that levels of 37.2% ± 10.0% and 100.0% ± 0.0% respectively were determined, (Fig. 3).

### Separating the anterior and posterior capsules to create open capsular bag cultures retards cell cover on the posterior capsule of human capsular bags

**Figure 4:**
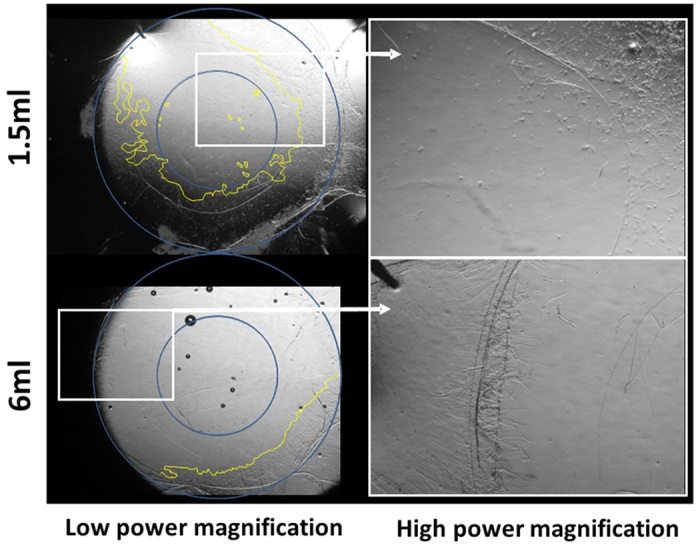
Capsule separation decreases cell growth: Comparison of 1.5 ml and 6 ml SF EMEM treated match-paired open capsular bags cultured for 28 days. Phase micrographs at end-point (day 28) showing limited cell cover on the central posterior capsule (as marked by yellow line) in 1.5 ml open bag cultures and no cell cover in 6 ml counterparts. The location of the anterior rhexis was super-imposed. Cell cover past the super-imposed anterior rhexis edge indicates there was no significant difference in cell cover between the 1.5 ml and 6 ml open capsular bags groups. Replicate match-paired experiments *n* = 3. Average age of donor was 71.0 ± 3.1 years.

Match-paired open capsular bags maintained in 1.5 ml SF EMEM had very little cell coverage of the central posterior capsule region at endpoint (Day 28; Fig. 4). In fact cell cover was only observed in one (of three) open capsular bag maintained in 1.5 ml SF EMEM (Fig. 4). No cell coverage was observed in the central posterior capsule region in open capsular bags maintained in 6 mls SF EMEM (Fig. 4).

Open match-paired capsular bags were also maintained in 1.5 ml and 6 ml 5%FCS supplemented EMEM. Complete coverage of the central posterior capsule region was obtained by day 12 of culture in both 1.5 ml and 6 ml counter parts (data not shown). This suggests that the reduction in cell cover observed in SF EMEM open-bag cultures can be negated by the addition of exogenous cytokines/growth factors (within serum).

### Cytokine analysis by 27-plex Bioplex identifies cytokines/growth factors that are significantly reduced in the bathing media of 6 ml capsular bag cultures compared to 1.5 ml

**Figure 5:**
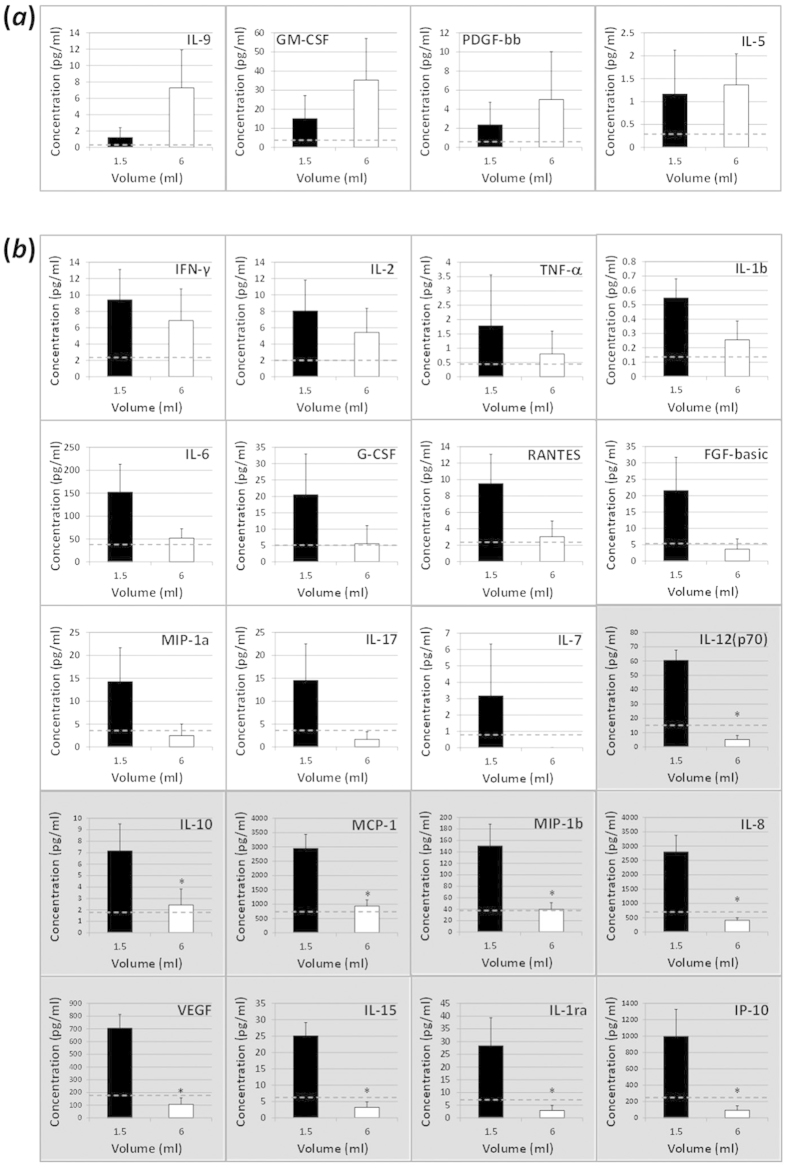
Comparison of growth factor/cytokine levels in 1.5 ml and 6 ml SF EMEM treated match-paired capsular bags at day 2 of culture. (**a**) Shows cytokines that increased in concentration in response to medium dilution and (**b**) shows those that were lower in 6 ml cultures relative to 1.5 ml counterparts. Cytokine levels in media of closed match-paired capsular bag cultures maintained in SF EMEM was analysed by a suspended bead array multiplex cytokine panel. Data expressed as Mean ± SEM (*n* = 10). *Indicates significant difference between the 1.5 ml and 6 ml groups (P ≤ 0.05, Students t test). Average age of donor was 72.8 ± 2.7 years.

Analysis of media collected from closed capsular bag preparations at culture day 2 demonstrated that 24 cytokines were detectable in bathing media from both 1.5 ml and 6 ml cultures (Fig. 5). While one would predict that all cytokines would be diluted four fold with increasing culture medium volume it is important to note that this is a biological system that is capable of reacting to its changing environment. Consequently only 9 cytokines (IL-8, IL-15, IL-12(p70), MCP-1, MIP-1β, IL-1ra, IL-10, IP-10 and VEGF) were found to have significantly reduced levels in the media of capsular bags maintained in 6 mls SF compared to 1.5 ml (Fig. 5). This pattern correlates with growth across the capsular bag and as a result these 9 candidates were studied further to assess their ability to stimulate survival and growth.

### Stimulation of FHL 124 cell growth by cytokine candidates

**Figure 6:**
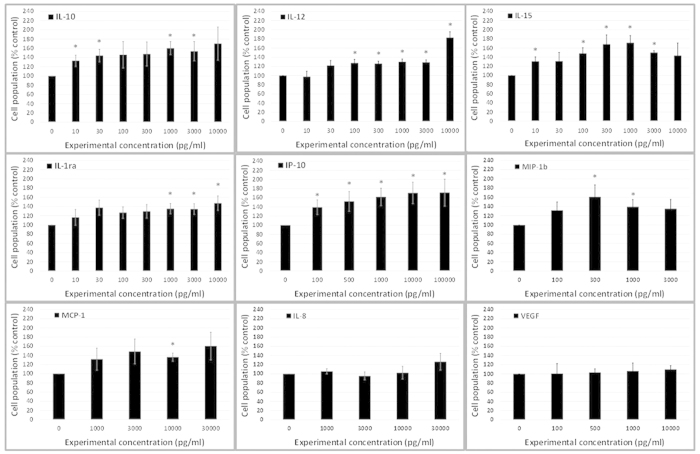
Cytokine addition increases cell proliferation: Specific cytokine addition to human lens cell line FHL 124 cells over a 48 hr period increases cell proliferation. No increase in cell proliferation was observed with concentrations of IL-8 and VEGF. Cell proliferation detected by alcohol extraction of Coomassie blue protein dye. The data are expressed as mean ± SEM (*n* = > 4). *Indicates significant difference to control (P ≤ 0.05, Students t test).

The addition of IL-10, IL-12, IL-15, IL-1ra, IP-10, MCP-1 or MIP1β to the human lens cell line FHL 124 cultured in SF EMEM resulted in significant cell population increases after 48 hours relative to non-stimulated controls (Fig. 6). Generally, this response occurred in a dose-dependent manner. However, the addition of IL-8 and VEGF did not result in a change in the cell population. This outcome is particularly interesting in relation to VEGF because it is highly expressed in the bathing media of 1.5 ml capsular bag cultures (Fig. 5) and raises the possibility that endogenous VEGF could be a key survival/growth factor.

### VEGF receptor expression in human lens cells

**Figure 7:**
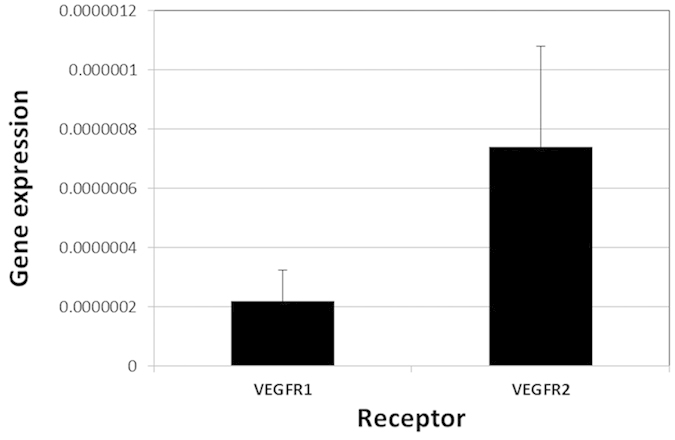
Quantitative comparison of VEGFR1/Flt-1 and VEGFR2/KDR gene expression in FHL 124 cells. The y-axis represents the gene of interest/18S expression calculated as mean ± SEM (n = 4).

The expression of VEGFR1 and VEGFR2, also known as flt1 and KDR respectively, were determine in FHL 124 cells using real-time PCR. Both receptors were detectable, but the signal for VEGFR2 was consistently greater (Fig. 7). These data demonstrating the presence of VEGF receptors allied to detection of VEGF in the culture medium indicates that VEGF/VEGFR autocrine signalling can take place.

### Inhibition of VEGF receptors reduces cell viability, growth and transdifferentiation of FHL 124 cells

**Figure 8:**
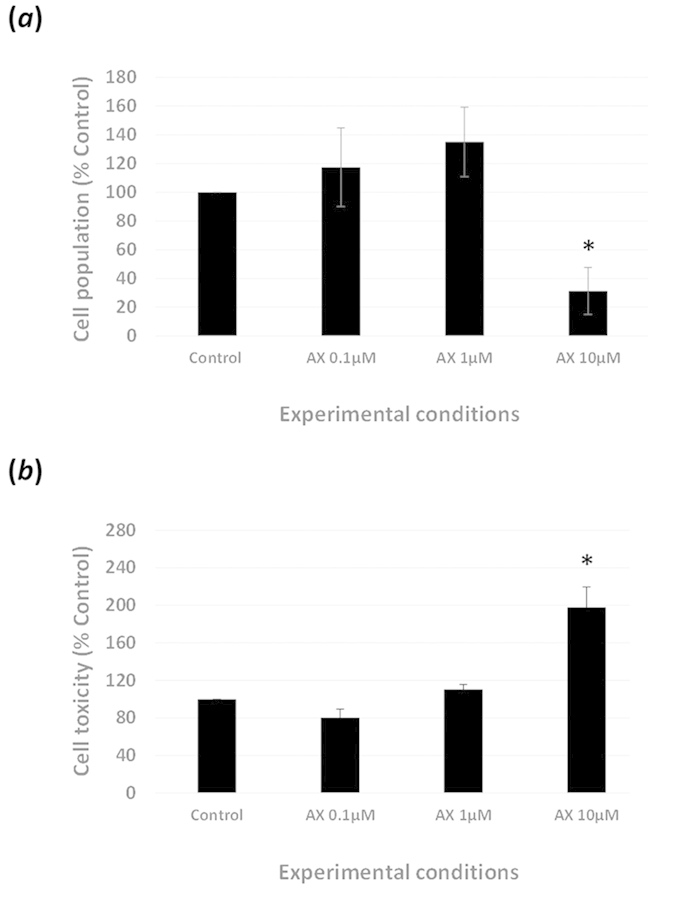
VEGF receptor inhibition modulates cell population and toxicity: (**a**) Addition of Axitinib (10 μm) to FHL 124 cells cultured in SF EMEM over a 72 hr period caused a significant decrease in cell population. (**a**) No significant changes in cell populations were found with exposure to 0.1 and 1 μM Axitinib compared to control. (**b**) A significant increase in cell toxicity was observed with the addition of 10 μM Axitinib but not with exposure to 0.1 and 1 μM Axitinib. Cell population detected by alcohol extraction of Coomassie blue protein dye. Cell toxicity assessed by total LDH using commercially available kit (Promega). The data are expressed as mean ± SEM (*n* = 3). *Indicates significant difference to control (P ≤ 0.05, Students t test).

**Figure 9:**
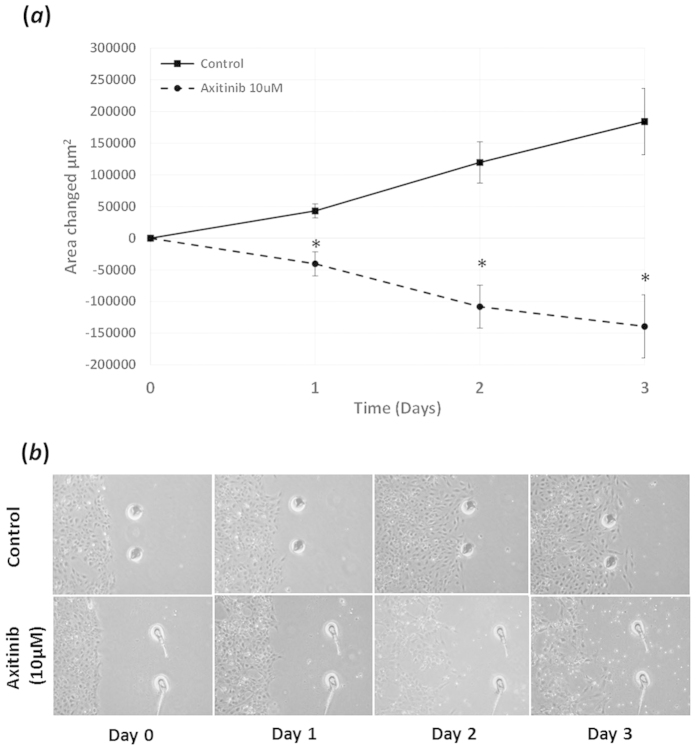
VEGF receptor inhibition affects cell migration: (**a**) Addition of Axitinib (10 μM) to FHL 124 cells cultured in SF EMEM over a 3 day period caused a significant decrease in cell migration compared to SF Controls. (**a**) This decrease was less than control conditions indicating that Axitinib (10 μM) was additionally affecting cell survival. (**b**) Cell migration was determined using a scratch-wound healing assay and measuring cell movement from the leading edge with Image J analysis software. The data are expressed as mean ± SEM (*n* = 3). *Indicates significant difference to SF Control (P ≤ 0.05, Students t test).

**Figure 10:**
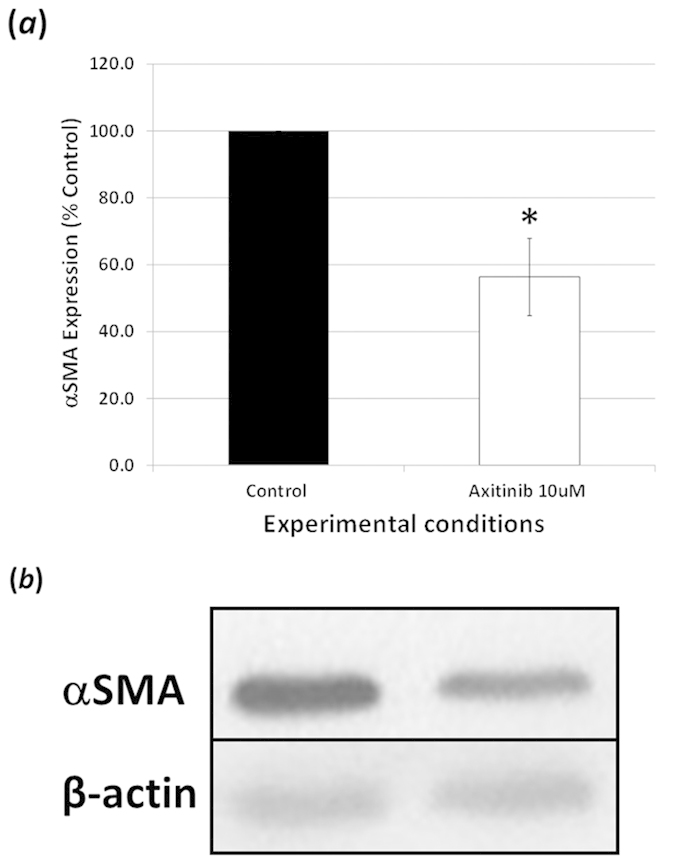
Alpha smooth muscle actin expression: (**a,b**) Axitinib (10 μM) decreased protein levels of the myofibroblastic marker αSMA in FHL 124 cells. FHL 124 cells were treated with either Axitinib (10 μM) or SF EMEM for 48 hrs following which protein was extracted. (**a,b**) Western blot analysis shows a significant decrease in αSMA in FHL 124 cells exposed to Axitinib (10 μM) for 48 hrs compared to SF EMEM treated controls. (**a,b**) Data were normalised to β-Actin control. Data are expressed as Mean ± SEM (*n* = 3). *Indicates significant difference between the Control and Axitinib (10 μM) groups (P ≤ 0.05, Students t test). Samples for each experiment were run on the same gel and processed in parallel. All gels were run under the same experimental conditions. The representative western blots are cropped images and full-length blots are presented in Supplementary Figure 1.

**Figure 11:**
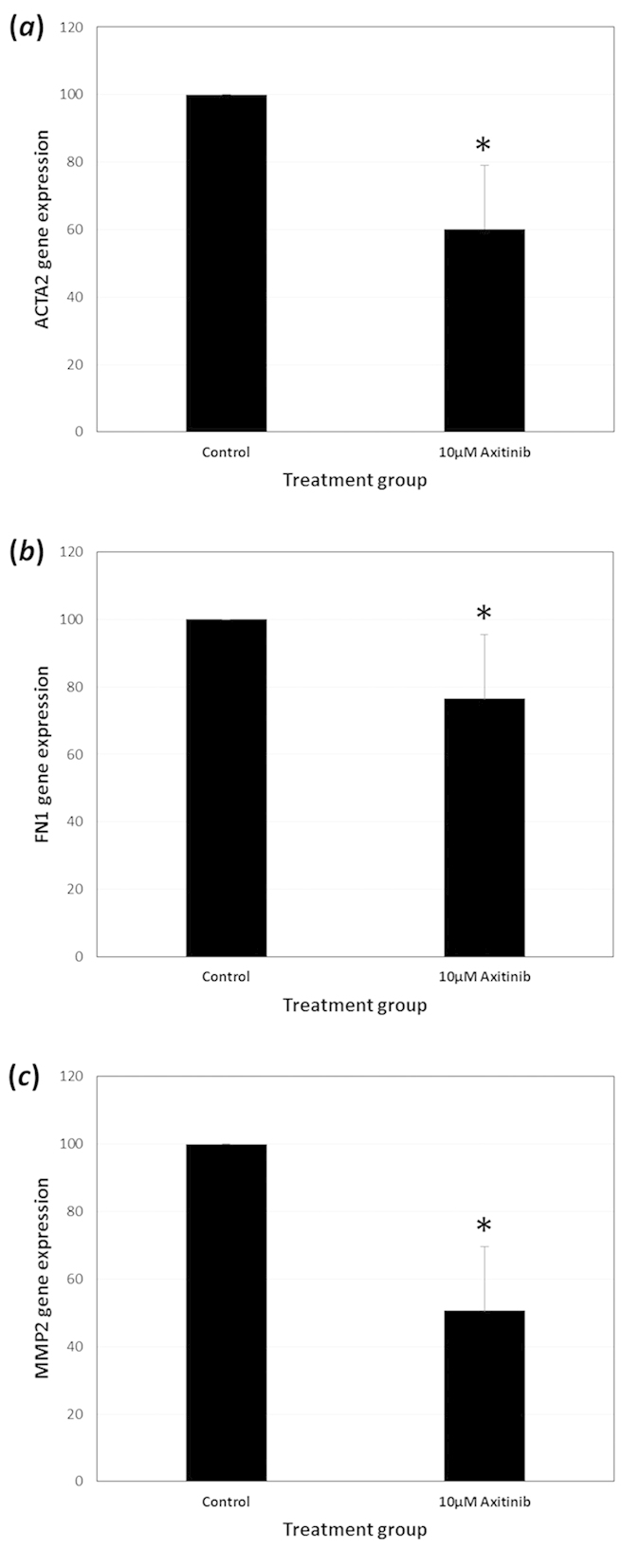
The influence of VEGF receptor inhibition using Axitinib (10 μM) on EMT/fibrotic marker genes (**a**) ACTA2 (**b**) FN1 and (**c**) MMP2 in FHL 124 cells. Cells were maintained in experimental conditions for 24 hours and gene expression was detected using TaqMan real-time PCR. Expression of the gene of interest was normalised to 18S expression. The data represent Mean ± SEM (*n* = 3). *indicates a significant difference between treatment and control group ((P ≤ 0.05, Students t test).

Treatment with the pan-specific VEGF receptor inhibitor Axitinib at 10 μM lead to a significant reduction in cell population following a 72 hour culture period (Fig. 8a). No significant changes were observed with lower concentrations (0.1 and 1 μM). To see if the reduction in cell population associated with 10 μM treatment corresponded with increase cell death the LDH assay was employed. This analysis demonstrated no greater loss in LDH with 0.1 or 1 μM Axitinib relative to control, but a significant increase was observed with 10 μM (Fig. 8b). To better observe the changes taking place within the 72 hour culture period and further assess the impact of VEGFR inhibition a scratch wound-healing assay was used. Employing this method demonstrated that after 24 hours of culture, a significant decrease in cell migration was detected when comparing Axitinib (10 μM) treated cells to SF controls (Fig. 9). At this stage the cells look generally healthy, but as the period of culture extended to 48 hours some evidence of cell death was apparent in the treated group whereas migration progressed in the control cells. This pattern continued to end-point (72 hours) when notable distress to cells with Axitinib treatment had occurred (Fig. 9). The effect of VEGFR inhibition on transdifferentiation was also assessed using the myofibroblast cell marker, αSMA as a measure. Addition of Axitinib (10 μM) to FHL 124 cells for 48 hrs significantly decreased the protein levels of αSMA to 56.4 ± 11.5% (Fig. 10) of non- treated control cells. This EMT response was also examined at the message level using real-time PCR. Using this method it was found that ACTA2, the gene encoding αSMA was significantly reduced with Axitinib treatment. In addition, EMT/fibrosis associated genes, FN1 and MMP2 gene expression was also significantly suppressed with VEGFR inhibition (Fig. 11). This collection of data support the notion that VEGF is important for cell survival, growth and transdifferentiation of human lens cells.

### VEGFR inhibition reduces PCO related events in a human *in vitro* capsular bag culture system

**Figure 12:**
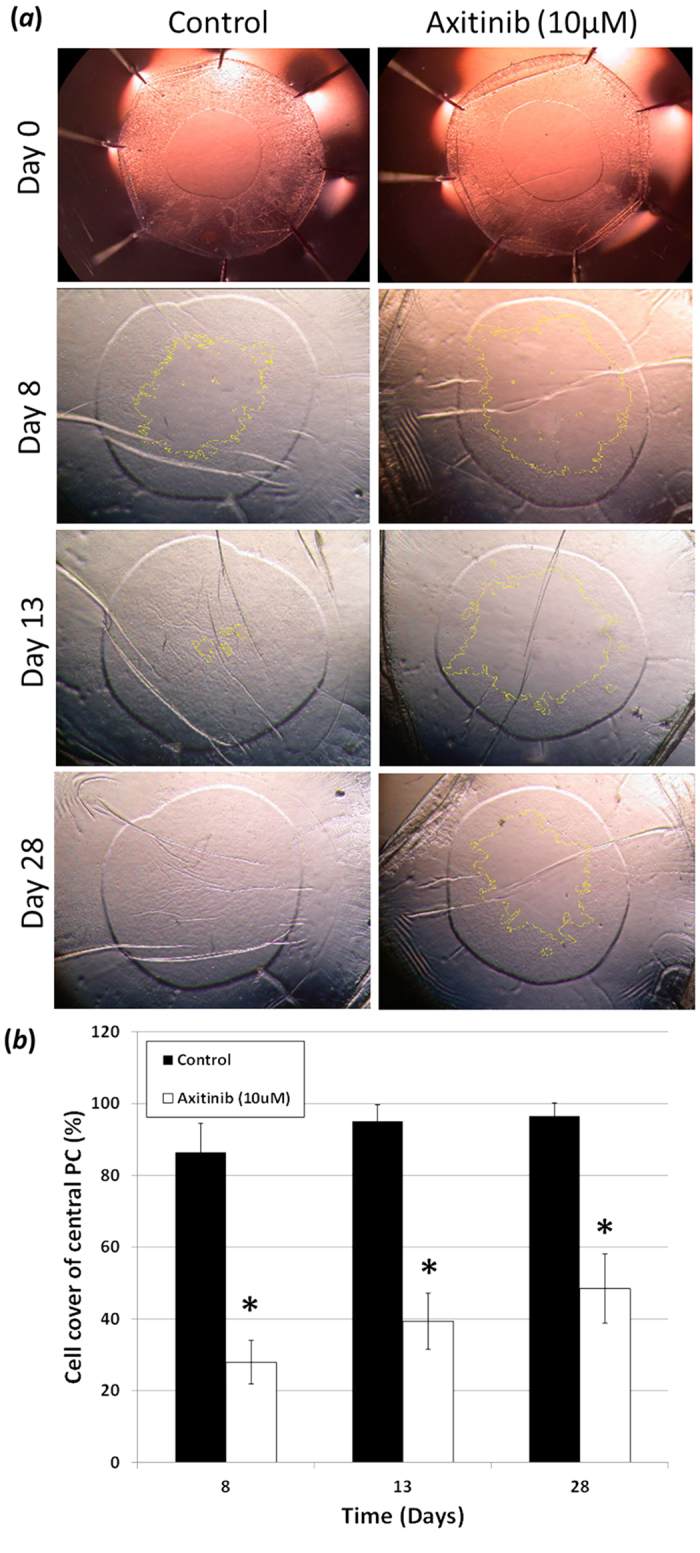
Posterior capsule cell cover: (**a,b**) Comparison of SF EMEM Control Vs Axitinib (10 μM) treated match-paired capsular bags cultured over a 28 day period. (**a**) Phase micrographs at day 0 showing no cell cover on the central posterior capsule viewed via the anterior capsule rhexis. (**a**) Cell progression has advanced onto the central posterior capsule region at day 8 of culture in both Control and Axitinib (10 μM) treated cultures. (**b**) This cell cover is significantly different between the match-paired counterparts. (**a,b**) Indicating a significantly greater cell cover on the central posterior capsule in Control cultures compared to Axitinib (10 μM) treated counter-parts at day 13 of culture. (**a,b**) Day 28 (end-point) of culture indicating almost complete cell cover of the central posterior capsule in Control capsular bags compared with a significant reduction in cell cover in Axitinib (10 μM) treated counterparts. Data are expressed as Mean ± SEM (*n* = 5). *Indicates significant difference between the Control and Axitinib (10 μM) groups (P ≤ 0.05, Students t test). Average age of donor was 63.0 ± 5.0 years.

**Figure 13:**
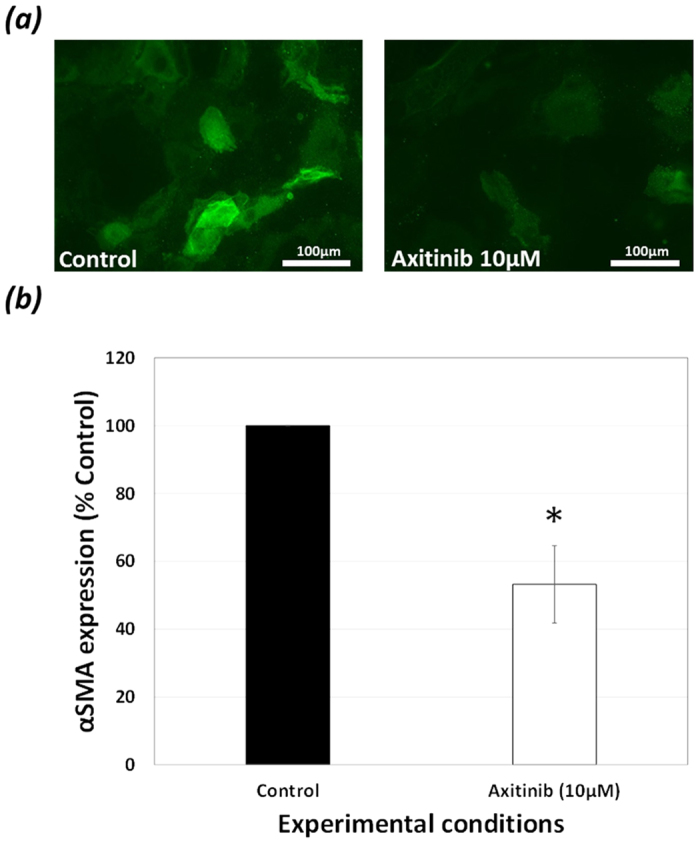
EMT decreases with VEGFR inhibition: (**a,b**) Comparison of the expression of myofibroblastic cell marker αSMA in Control and Axitinib (10 μM) SF EMEM treated match-paired capsular bags cultured for 28-days. (**a,b**) Epifluorescence micrographs at day 28 show a significant decrease in αSMA expression in cells on the central posterior capsule in Axitinib treated cultures compared to Control counter-parts. The data are expressed as Mean ± SEM (*n* = 5). *Indicates significant difference between the Control and Axitinib groups (P ≤ 0.05, Students t test). αSMA expression shown in green.

There was no significant difference in the point at which cells passed the rhexis edge on to the central posterior capsule in capsular bags cultured in Axitinib (10 μM) 1.8 ± 0.2 days compared to control counterparts 1.6 ± 0.2 days. However, a significant difference could be observed by day 8 in cell cover of the central posterior capsule region with Axitinib (10 μM) treatment compared to controls, such that coverage in these two groups was 27.9 ± 6.1% and 86.4 ± 8.1% respectively (Fig. 12). This difference in cell cover was retained throughout the culture period with a significant difference still observed at day 13 of culture, with 95.1 ± 4.6% cover seen in control bags and 39.3 ± 7.8% for Axitinib (10 μM) treated counterparts (Fig. 12). At endpoint (Day 28) the control capsular bags had almost complete cell coverage of the central posterior capsule region with an average of 96.5 ± 3.5%, whilst the Axitinib (10 μM) treated counterparts had a significantly reduced cell cover of 48.5 ± 8.0% (Fig. 12). Further evaluation at end-point to assess levels of αSMA in cells growing on the central posterior capsule, demonstrated a significant reduction in transdifferentiation with VEGFR inhibition relative to non-treated controls, such that levels of 53.2% ± 11.4% and 100.0% ± 0.0% respectively were determined, (Fig. 13).

## Discussion

The development of open bag IOL devices has opened up a new avenue in the management of PCO following cataract surgery. The separation of the anterior and posterior lens capsules is believed to allow greater irrigation of the capsular bag, which in turn hypothetically reduces cytokine/growth factor enrichment to remaining lens cells. This reduced supply of stimuli is predicted to limit the rate of PCO progression. In the current study we demonstrate that dilution of cytokines/growth factors by increased culture medium volume, to mimick greater irrigation, significantly retards growth across the previously cell free posterior capsule and leads to reduced myofibroblast formation. Attenuation of these events support the notion that open bag IOLs can provide benefit to cataract patients through restriction of PCO. In addition, we identified that expression of several cytokines/growth factors correlated with cell growth following simulated cataract surgery on donor eyes. Of these we determined that endogenous VEGF in particular played an important role in lens cell survival, growth and transdifferentiation and thus provides a therapeutic target to further manage PCO development in conjunction with improved IOL designs.

The IOL is an important component in the management of PCO following cataract surgery. At present a square edge acrylic IOL with a 360 degree contact of the anterior capsulorhexis on the optic body is regarded as best practice^12^,^13^. This strategy allows the anterior and posterior capsules to come in contact and through a cell mediated process these surfaces are fused, which in turn ‘shrink wraps’ the IOL within the capsular bag. The forces exerted through this process increase tension of the capsule against the square edge of the optic and this forms a physical barrier that impedes the progression of cells across the visual axis^14^. Adoption of the square-edge IOL implant has reduced PCO, but in time this barrier is overcome and cells progress onto the posterior capsule leading to a reduction in visual quality. Square edge IOLs afford some benefit to patients, but there is still considerable scope to develop IOLs that better contain PCO. Open bag designs are beginning to emerge^15^,^8^ and early reports both in pre-clinical and clinical scenarios suggest that this approach is effective^16^,^17^. It is hypothesised that greater irrigation of the capsular bag will limit cytokines/growth factor availability to lens cells, which in turn will limit PCO progression. Previously, we have shown that an open bag IOL is more effective at reducing cell progression on to the posterior capsule in comparison to a leading square-edge closed bag IOL^9^. Our findings support this idea. In our study, to mimick greater dilution of growth factors that is predicted with open bag IOLs, we simply cultured cells in different culture medium volumes. These were closed bags without an IOL implanted and interesting results were obtained. First of all the time at which cells appeared on the central posterior capsule, beyond the capsulorhexis margin of match-paired cultures did not differ, suggesting that a lack of irrigation in the closed compartment rendered growth in these closed environments independent of total culture volume. Once cells reached the exposed central posterior capsule the greater culture volume took effect and growth was restricted and interestingly at end-point, transdifferentiation was also reduced. These findings suggest that the proximity of the anterior and posterior capsules is important for cell progression. It is likely that the cells present in this region provide adequate enrichment of growth factors to neighbouring cells and it is also likely that adsorption of growth factors to matrix components within the lens capsule will accrue and provide an available resource. Interestingly when the capsular bag was fully opened, such that all internal surfaces were laid flat and directly exposed to culture medium, cell growth onto the posterior capsule was significantly restricted. This finding ties in with the idea that within a closed system cytokine/growth factor production is better able to support the cell populations. In the fully open system lower concentrations are likely to result and potentially, matrix bound growth factors will be less. Exposure of multiple cytokines/growth factors by serum addition promoted growth across the posterior capsule, which again supports the idea that a shortfall in stimuli is a rate limiting factor. Having demonstrated the restriction of growth and transdifferentiation through irrigation and separation of the lens capsules it is important to consider, which cytokines/growth factors are regulating events following surgery? In order to assess multiple cytokines/growth factors we employed a suspended bead cytokine array analysis of medium samples. The capsular bag system is dynamic and responsive to its environment. It was, therefore, uncertain whether culture in a four-fold greater medium volume would result in a quarter of the level of growth factors. Consequently, we needed to identify which cytokines/growth factors were present and how they were modified by a change in culture medium volume. In some cases, levels actually went up with greater culture volume suggesting a cellular response to the situation. However, we were particularly interested in the factors that showed a significant decrease in levels with greater culture volume, which would correlate with PCO related events. We found nine candidates using this screen. These were IL-1ra, IL-8, IL-10, IL-12(p70), IL-15, IP-10, MCP-1, MIP-1β and VEGF. A number of these cytokines have been linked to cell growth and other fibrotic conditions^18^,^19^. IL-10, while not directly linked to proliferation does demonstrate a correlation in expression with VEGF levels and it is proposed that IL-10 mediates VEGF expression^20^. Similarly, MCP-1 is also reported to regulate proliferation through endogenous VEGF^21^. This could be through increased synthesis or greater liberation of VEGF from matrix associated stores. VEGF is known to bind to heparan sulphate proteoglycans (HSPGs)^22^, which are abundant on the lens capsule^23^,^24^. Therefore, increased levels of VEGF as a consequence of cataract surgery could bind to the capsule and provide a sustained supply of VEGF that can mediate long-term changes. VEGF can be released from extracellular matrix by plasmin cleavage^25^ or MMPs^26^. Our previous studies have demonstrated that MMPs increase in culture media following cataract removal surgery^27^,^28^ and thus could perform this function. In the lens capsular bag model, levels of MMPs are known to rise and thus it is feasible that this could be linked to specific cytokines. Using cell culture systems we demonstrated that exogenous application of all, but IL-8 and VEGF were capable of promoting further lens epithelial cell growth. With respect to IL-8 and VEGF, it should be noted that both of these candidates were expressed at relatively high levels in 1.5 ml cultures and thus it is possible that they contribute greatly to endogenous maintenance of lens epithelial cells. As a consequence we decided to adopt an inhibition strategy and elected to concentrate our efforts on VEGF because there are established inhibitors^29^,^30^,^31^ and a range of therapeutic agents commonly used in the clinic to treat the wet form of age-related macular degeneration (AMD)^32^. Using this approach we found that VEGFR inhibition could significantly retard growth and restrict myofibroblast formation.

We hypothesised that endogenous VEGF was a survival/growth factor for human lens epithelial cells.

This theory was supported by the inhibition study data as cell growth was suppressed in both cell line and capsular bag experiments. It was also apparent that cell death was observed over time, which was more apparent in the cell line than capsular bags, which could suggest that differences in matrix composition or cell density could be important factors. As PCO is a fibrotic condition, the effects of VEGF inhibition on myofibroblast formation is of interest. Our findings demonstrated that VEGFR inhibition could significantly decrease αSMA, a myofibroblast marker, in both the cell line and in capsular bags. This pattern of response also compares to the results obtained for capsular bags cultured in greater media volume. These studies were performed to investigate modification of baseline levels of αSMA in response to surgical injury. It is likely that TGFβ levels will rise in the eye^33^ and that exposure of lens cells to this stimulus will increase myofibroblast numbers^34^,^35^,^36^. It will therefore be of great interest in the future to test the effects of VEGF inhibition on TGFβ-induced myofibroblast formation.

It should be noted that the current study employed pan-specific VEGFR inhibitor (Axitinib). While this served as an excellent tool to identify the key roles of VEGF in PCO related events it does not allow analysis of the relative contributions of VEGFR sub-types. Future studies could utilise more selective inhibitors against individual VEGFRs or if required siRNA knockdown. One possibility is to apply the inhibitor to the aqueous or vitreous humour at the time of surgery. This approach, however, is dependent on adverse effects on non-lenticular tissue. An alternative strategies is to deliver the drug to lens cells through localised drug delivery^36^,^37^,^38^,^39^. This can be achieved by modification of the intraocular lens surface^36^,^40^,^41^,^42^, however, important consideration is required to reduce the effect of abrasion of the surface when implanted through a narrow injection device. These lenses are folded prior to implantation and coating an inner surface could be preferable. While this principle applies to any IOL based on our findings it would be most effect if an open bag IOL is used. Another method that provides local delivery to lens cells is the perfect capsule system^37^,^39^,^43^. This clinical tool provides a seal over the capsulorhexis. Through an irrigation port a drug can be introduced for a designated period of time and removed through an aspiration port. This approach allows controlled delivery and can be used in any cataract surgery independent of which IOL is implanted. While VEGFR inhibitors provide a therapeutic option it should be noted that several therapies for wet AMD target VEGF with the majority being antibody based^43^. Again similar modes of delivery, as described for the inhibitors could be used. These therapeutic agents have the benefit of being FDA approved for use in the eye and thus could be adopted for use in cataract patients without major delay.

## Conclusions

The data elucidated from this project adds to the body of data revealing that changes in IOL design to an “open bag” device could substantially reduce the rate of PCO development. Here open capsular bag *in vitro* cultures showed a significant decrease in cell cover of the posterior capsule compared to closed capsular bag preparations. This supports the idea that improved irrigation using open bag IOLs limits growth factor availability and suppresses PCO like events, such as cell growth and myofibroblast expression. Further adaptation to open bag designs by modification of open bag IOL devices with inhibitors to VEGF could further enhance the inhibitory effect on cell progression to the posterior capsule following cataract removal. Prevention of PCO using this method will maintain a greater level of visual quality, reduce the need for secondary surgery and potential provide a marked financial benefit to healthcare providers.

## Materials and Methods

All reagents were from Sigma-Aldrich (Poole, Dorset, UK) unless otherwise stated.

### Capsular bag preparations

**Figure 14:**
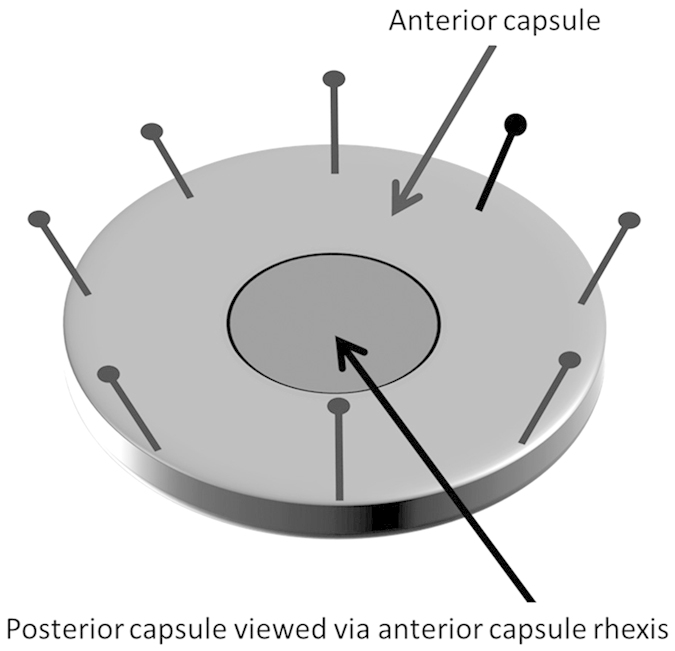
Diagram of closed capsular bag preparation from a human donor lens.

Cataract operations were performed on human donor lenses to create capsular bags from that were obtained with informed consent and used in accordance with the tenets of the Declaration of Helsinki. Approval for the study and experimental protocols (04/Q0102/57) was granted by a national research ethics committee under the Health Research Authority (UK). Relevant information relating to the donors is provided in the Supplementary data section (Table 1). A small capsulorhexis was made in the centre of the anterior lens capsule through which the fibre-cell mass of the lens was removed by hydro-expression. This created the capsular bag consisting of the entire “cell-free” posterior capsule and a ring of anterior capsule with its associated anterior lens epithelial cells. The capsular bag was then dissected from the globe by cutting through the zonules of Zinn. The capsular bag was then pinned onto 35 mm petri dishes (Corning Incorporated Life Sciences, New York, USA) using entomological pins (Anglia Lepidopterist supplies, Hindolveston, Norfolk, UK). This resulted in a closed capsular bag preparation (Fig. 14). All capsular bags contained similar starting populations of anterior lens epithelial cells.

Capsular bags were subsequently maintained in either non-supplemented SF EMEM (Eagles, minimum essential medium) containing Gentamicin antibiotic (50 μg/mL) at a volume of 1.5 ml or 6 mls or SF EMEM containing Gentamicin antibiotic (50 μg/mL) ± Axitinib (10 μM) (Selleckchem (Stratech Scientific Ltd), Newmarket, Suffolk, UK)). Capsular bag preparations were maintained for 28 days in a 35 °C, 5% CO_2_ incubator and experimental conditions replenished every 2–3 days. Ongoing observations were performed using a Nikon Eclipse TE200 phase-contrast microscope and phase contrast images taken. End point analysis of cell coverage on the previously cell-free posterior capsule was assessed with Image J analysis software (http://rsb.info.nih.gov/ij/), using the area of the anterior capsulorhexis formed during the cataract operation as a control. Complete cell coverage of the posterior capsule as observed thorough the created anterior capsulorhexis was scored as 100% cell coverage, the effect of experimental conditions on the progression of lens epithelial cells onto the posterior capsule was evaluated.

**Figure 15:**
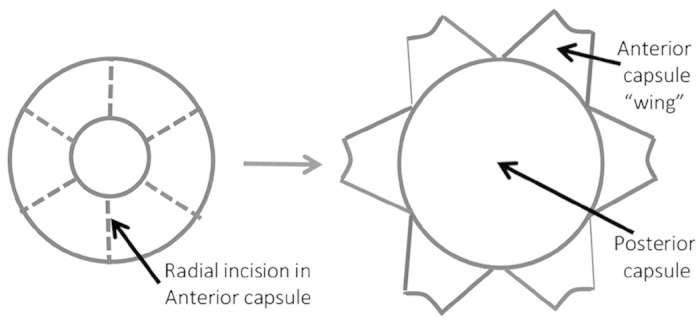
Diagram of open capsular bag preparation from a human donor lens.

Open bag preparations were created as above but following creation of the anterior capsulorhexis radial incisions were made in the anterior capsule and the resultant anterior capsule “wings” pinned open (Fig. 15). The fibre-cell mass of the lens was removed and the preparations cultured in either 1.5 ml or 6 ml SF EMEM containing Gentamicin antibiotic (50 μg/mL) for 28 days. Ongoing observations were performed using a Nikon Eclipse TE200 phase-contrast microscope and phase contrast images taken. End-point analysis of cell coverage on the previously cell-free posterior capsule was assessed with Image J analysis software (http://rsb.info.nih.gov/ij/), the area of the anterior capsulorhexis was super-imposed on the posterior capsule image and cell coverage of the posterior capsule measured within the super-imposed rhexis region. Complete cell coverage of this area was scored as 100% cell coverage, the effect of media volume increase on the progression of anterior lens epithelial cells onto the posterior capsule was evaluated.

### FHL 124 cells

The foetal human lens epithelial cell-line FHL 124 used in this study has previously been shown to have 99.5% homology with native human lens epithelium^44^,^45^. FHL 124 cells were kindly provided by Prof. J. R. Reddan (Oakland University, Rochester, MI).

### Cell proliferation

FHL 124 cells were seeded onto 96 well mictotitre plates (Thermoscientific, Cramlington, Northumberland, UK) at a density of 2500 cells per well and maintained for 3 days in 5% FCS EMEM supplemented with Gentamicin antibiotic (50 μg/mL) in a 35 °C 5% CO_2_ incubator before 24 hr serum starvation. Media was then aspirated and replaced with increasing dose response of the following cytokines IL-8, IL-15, IL-12(p70), MCP-1, MIP-1β, IL-1ra, IL-10, IP-10 and VEGF for 48 hrs. At experimental end points media was aspirated and cells fixed with 4% formaldehyde in PBS (Oxoid Ltd, Basingstoke, Hampshire, UK) for 30 minutes, formaldehyde was subsequently removed and cells washed with PBS 3 times for 5 minutes. To each well 200 μl of Coomassie blue protein stain (1 g Coomassie brilliant blue G (Merck, Darmstadt, Germany), 200 ml methanol, 40 ml glacial acetic acid (Fisher, Scientific UK Ltd, Loughborough, UK), 100 ml dH_2_O) was added for 30 minutes and the plate placed on a microtitre plate shaker (Heidolph instruments, Schwabach, Germany). Coomassie blue stain non-specifically binds proteins and can therefore be used to determine cell population numbers. After 30 minutes, the Coomassie blue stain was removed by several washes in PBS. To each well 200 μl of 70% ethanol was added and the plate placed on a microtitre plate shaker (Heidolph instruments, Schwabach, Germany) for 30 minutes to elute the bound dye. The plate was then read at 550 nm absorbance on a BMG Fluostar spectrophotometer plate reader (BMG Labtech, Aylesbury UK). Data was analysed as a percentage change in absorbance at 550 nm from untreated samples.

### Cell toxicity

FHL 124 cells were seeded onto 96 well mictotitre plates (Thermoscientific, Cramlington, Northumberland, UK) at a density of 2500 cells per well. At experimental end-points media was collected and 100 μl of each sample added to a well on a new 96 well mictotitre plates (Thermoscientific, Cramlington, Northumberland, UK). To determine LDH content in the cells 100 μl of 2% Triton X-100 in PBS was added to each well to lyse the cells and the plate placed on a microtire plate shaker (Heidolph instruments, Schwabach, Germany) for 30 minutes. Following this time 10 μl of the lysate was transferred to wells on a separate microtitre plate and 90 μl of SF EMEM added. A non-radioactive cytotoxicity assay (Roche, Germany) was used to measure the amount of lactate dehydrogenase (LDH) in both the sampled media and the lysate. The procedure was in accordance with the manufacturer’s protocol. The absorbance of all samples was recorded at 490 nm using a BMG Fluostar spectrophotometer plate reader (BMG Labtech, Aylesbury UK). The percentage of total LDH released into the culture medium was calculated using the following formula media/(media + lysateX10)*100.

### Scratch wound-healing assay

FHL 124 cells were seeded on to 35 mm tissue culture dishes (Corning Incorporated Life Sciences, New York, USA) at a density of 50000 cells/dish and maintained in SF EMEM supplemented with 5% FCS and Gentamicin antibiotic (50 μg/mL) until a confluent region spanning approximately 1 cm developed. Media was then aspirated and replaced with non-supplemented SF EMEM with Gentamicin antibiotic (50 μg/mL) and cultured for a further 24 hrs. Subsequently, a scratch was made through the centre of the confluent sheet using a plastic pipette tip and the cells removed from one half of the dish using a cell scraper. Orientation marks were made near the wound edge to create a reference point. Ongoing observations were performed using a Nikon Eclipse TE200 phase-contrast microscope and phase contrast images taken. The change in area covered over time in each experimental condition was calculated from the phase contrast images using Fiji (Fiji Is Just Image J) Image J analysis software (http://fiji.sc/Fiji).

### Western blotting

Following culture of FHL 124 cells in SF EMEM or Axitinib (10 μM, Selleckchem (Stratech Scientific Ltd), Newmarket, Suffolk, UK)) for 48 hrs cells were immediately quickly washed with ice-cold PBS (phosphate buffered saline). Ice-cold PBS was aspirated and replaced with 350 μl of MPER lysis buffer (Thermoscientific, Cramlington, Northumberland, UK) containing 5 mM EDTA and 10 μl/mL of both protease and phosphotase inhibitor cocktails (Thermoscientific, Cramlington, Northumberland, UK). Following a 5 minute incubation on ice, cells were detached from dishes using a cell-scraper and the resultant lysates carefully collected and transferred to 1.5 ml Eppendorf tubes and centrifuged for 10 minutes at 1300λPM. Soluble fractions were transferred to fresh 0.5 ml Eppendorfs tubes and stored at −20 °C, whilst the insoluble fractions were discarded.

To ensure an equal quantity of protein for each sample was loaded on to SDS-PAGE gels a BCA (Bicinchronic) protein assay was performed. A protein standard series ranging from 0–1000 μg/ml was generated from a 2 mg/ml BSA in lysis buffer stock solution. Ten micro-litres of each standard and unknown sample were pipetted into individual wells of a transparent 96-well microtitre plate (Thermoscientific, Cramlington, Northumberland, UK), along with 40 μl of double distilled water. Protein standards were tested in triplicate, whilst all unknown samples were tested in duplicate. Working reagent (BCA™ protein assay kit, Thermoscientific, Cramlington, Northumberland, UK) at a volume of 200 μl was added to each well (concentration 1:50) and the plate was gently shaken on a microtitre plate shaker for 1 minute prior to a 1 hr incubation at 35 °C. Subsequently the plate was left at room temperature to cool for 5 minutes and then analyzed in a BMG Fluostar spectrophotometer plate reader (BMG Labtech, Aylesbury UK) at a setting at 565 nm. The protein concentration of each unknown sample could then be calculated from the standard dilution series. Equal amounts of protein from each sample were loaded onto 10% SDS-PAGE gels for electrophoresis, and then transferred onto PVDF membrane (NEN Life Science Products, Boston, MA, USA) using a Trans-Blot semi-dry Transfer Cell (Bio-Rad, Hemel Hempstead, Hertfordshire, UK). Subsequently the membrane was blocked with a PBS solution containing Tween (1:2000, Fisher Scientific UK Ltd, Loughborough, UK) and milk protein (0.5%) to block non-specific sites for 1 hour. Specific proteins were probed using Anti-αSMA (1:1000) and Anti-β-Actin (1:1000) Cell Signalling Technology, Hitchin, Hertfordshire, UK) overnight at 4 °C. Following the incubation period the membranes were washed a further 6 times for 5 minutes per wash and then incubated for a further hour in secondary HRP antibody (1:2000 GE Healthcare, Little Chalfont, Buckinghamshire, UK). After secondary antibody incubation the membranes were washed a further 5 times in PBS solution containing Tween (1:2000, Fisher Scientific UK Ltd, Loughborough, UK) and milk protein (0.5%) before a final wash in PBS containing tween (1:1000, Fisher Scientific UK Ltd, Loughborough, UK) without milk protein. The chemoluminescent ECL Prime was then added to the membrane (GE Healthcare, Little Chalfont, Buckinghamshire, UK) for 5 minutes in the dark, before the membrane was exposed to Hyperfilm ECL photographic paper (GE Healthcare, Little Chalfont, Buckinghamshire, UK) in a dark room. The photographic paper was developed using developing solution, and the developer halted with stop solution (Photosol, inc Hallandale, FL, USA), before the photographic paper was made transluscent with Fixer solution (ILFORD PHOTO, Knutsford, Cheshire, UK). The resultant protein bands on the photographic film were scanned on a flatbed scanner and measured using image J analysis software (http://rsb.info.nih.gov/ij/).

### Total RNA extraction and cDNA generation

Total RNA was extracted from FHL 124 cells (seeded at density of 50,000 cells in 5% FCS + EMEM) that were then replaced with SF-EMEM one day prior to treatment. Experimental conditions were then applied and the cells harvested for RNA following a 24 hour period, according to the manufacturers instructions for the RNeasy micro kits (Qiagen, West Sussex, UK). In the initial step, RLT buffer (containing β-mercaptoethanol) was added to Eppendorf tubes containing cell lysates. The samples were then homogenized through a 20 gauge needle (0.9 mm) and syringe. The remainder of the protocol was as described by the manufacturer and included a DNase step. RNA was quantified using a NanoDrop ND-1000 spectrophotometer (NanoDrop, Wilmington, DE). For each round of RNA extracted, the ratio of absorptions at 260/280 nm ranged from 1.8 to 2.2 (mean = 2.0). Where possible, total RNA was immediately used for cDNA generation or briefly stored for up to a week at −80 °C. Generation of cDNA was performed with Superscript II reverse transcriptase (Invitrogen, Paisley, UK) according to the reverse transcription (RT) protocols of the manufacturer using random primers (Promega, Southampton, UK).

### Quantitative Real time Polymerase Chain Reaction (QRT-PCR)

Real time PCR was used to quantitate target gene expression in FHL 124 cells relative to an endogenous control gene (18S). Oligonucleotide primers and fluorescence-labeled probes for fibrotic markers (ACTA2, FN-1 and MMP2) and VEGFR genes were ordered from Life Technologies (Supplementary Table 2). Assuming 100% efficiency in the RT reactions, either 1 or 5 ng cDNA was used in real-time PCR reactions performed using a real-time PCR machine (ABI7700; Applied Biosystems). Reagent-based assays (TaqMan Universal PCR Master Mix, No AmpErase® UNG; Applied Biosystems) containing all PCR reagents were employed according to the manufacturer’s instructions. Conditions for the PCR reaction were; 2 min at 50 °C, 10 min at 95 °C and then 40 cycles, each consisting of 15s at 95 °C and 1 min at 60 °C. The cycle number at which amplification entered the exponential phase (raw data cycle threshold [CT]) was determined and this number was used as an indicator for the amount of target RNA in each sample. In raw data analyses the CT value was used to classify gene expression as either very high (CT ≤ 22), high (CT = 23–25), moderate (CT = 26–28), low (CT = 29–34), or negligible to undetected (CT = 35–40). To determine the relative RNA levels in the samples, standard curves for each primer/probe set were prepared by using cDNA from one sample and making twofold serial dilutions covering the range equivalent to 20–0.625 ng RNA (for 18S analysis the range was from 1 to 0.03125 ng). Differences in the total amount of RNA present in each sample were normalized to endogenous 18S rRNA gene expression.

### Immunocytochemistry

Capsular bags were cultured in SF EMEM containing Gentamicin antibiotic (50 μg/mL) at a volume of 1.5 ml or 6 mls or SF EMEM containing Gentamicin antibiotic (50 μg/mL) ± (10 μM) Axitinib (Selleckchem (Stratech Scientific Ltd), Newmarket, Suffolk, UK)) maintained for 28 days. At end-point culture medium was aspirated from capsular bag culture dishes and the tissue washed 3 times in quick succession with PBS. Subsequently capsular bags were fixed in 1.5 ml of 4% formaldehyde in PBS solution for 30 minutes. The capsular bags were then bisected and each section transferred to a new 35 mm petri dish and washed for a further 3 times with a solution of PBS containing 0.02% Bovine Serum Albumen (BSA) and 0.05% Igepal. The cell membranes were permeabilised in PBS containing 0.5% Triton-X100 for 30 minutes. A further 3 washes in PBS containing 0.02% BSA and 0.05% Igepal were completed for a time period of 15 minutes per wash on a plate shaker. Non-specific cellular sites were blocked with Normal Goat Serum (1:50) in 1% BSA in PBS, and incubated for 1 hr at 37 °C. Following the blocking procedure 75μl of primary antibody for αSMA (1:100) was added to the tissue and capsular bags incubated for a further hour at 37 °C. Capsular bags were then washed for 15 minutes with shaking in PBS containing 0.02% BSA and 0.05% Igepal and this was repeated twice more. Secondary antibody (mouse anti-goat 488, Thermoscientific, Cramlington, Northumberland, UK) at a volume of 75 μl (1:200) was added to tissue and incubated for another hour at 37 °C in the dark. The Actin filament stain Texas Red X-Phalloidin (Thermoscientific, Cramlington, Northumberland, UK) 1:100 in PBS containing 1% BSA along with the chromatin stain DAPI (1:100) were added to preparations for ten minutes in the dark at room temperature. The capsular bags were washed a further three times in PBS containing 0.02% BSA and 0.05% Igepal with shaking in the dark for 10 minute durations. Capsular bags sections were mounted on microscope slides (Thermoscientific, Cramlington, Northumberland, UK) using Hydromount (National Diagnostics, Atlanta, Georgia, USA) mounting solution and left to dry for 30 minutes at room temperature in the dark and were subsequently viewed under a Zeiss CCD Upright epifluorescent microscope and analyzed using Axiovision 4.9.1 imaging software. Cell populations were quantified using Image J analysis (http://rsb.info.nih.gov/ij/) and αSMA expression levels with Image Pro Analysis software.

### Suspended Bead Array Analysis

Culture media was sampled on day 2 from capsular bag preparations maintained in either 1.5 ml or 6 ml SF EMEM. Media was analysed for cytokines IL-1β, IL-2, IL-4, IL-5, IL-6, IL-7, IL-8, IL-9, IL-10, IL-12(p70), IL-13, IL-15, IL-17, IL-1ra, VEGF, G-CSF, FGF (Basic), MIP-1α, MCP-1(MCAF), IP-10, RANTES, GM-CSF, IFNγ, TNFα, MIP-1β, Eotaxin and PDGF-BB using a commercially available 27-Plex panel (Bio-Plex Suspended Multiplex Bead Array Assay kit; Bio-Rad, Hemel Hempstead, UK) in accordance with manufacturer’s instructions. Data from the reactions were acquired with a flow cytometry system (X Map-100; Luminex, Austin, Texas, USA) and accompanying software (Bio-Plex Manager software; Bio-Rad). The median fluorescence intensity was used as a measure of detected protein.

### Statistical analysis

Statistical differences between groups were established using Student’s t-test with a p-value of ≤0.05 considered significant (Microsoft Excel).

## Additional Information

**How to cite this article**: Eldred, J. A. *et al.* Growth factor restriction impedes progression of wound healing following cataract surgery: identification of VEGF as a putative therapeutic target. *Sci. Rep.* **6**, 24453; doi: 10.1038/srep24453 (2016).

## Supplementary Material

Supplementary Information
