# Supplementary material for: Growth factor restriction impedes progression of wound healing following cataract surgery: identification of VEGF as a putative therapeutic target
Source: Sci Rep. 2016 Apr 14;6:24453. doi: 10.1038/srep24453 (PMC4831005; doi:10.1038/srep24453)
Supplement: Supplementary Information [file srep24453-s1.doc]

**Supplementary information**

**Growth factor restriction impedes progression of wound healing following cataract surgery: identification of VEGF as a putative therapeutic target**

Julie. A. Eldred, Matthew McDonald, Helen. S. Wilkes, David .J. Spalton, I. Michael Wormstone

**Supplementary Table 1**. Relevant information associated with human donors used in this study.

**Supplementary Table 2**. TaqMan assays employed to determine expression for genes of interest.

| **Gene** | ***Applied Biosystems* TaqMan Assay Number** | **Exon Spanning** | **NCBI Location Chromosome** | **NCBI Accession Number** |
| --- | --- | --- | --- | --- |
| ACTA2 | HS00426835_g1 | Yes | Chr.10: 90694831 - 90751147 | NM_001141945.1 |
| FN1 | HS00365052_m1 | Yes | Chr.2: 216225177 - 216300791 | NM_002026.2 |
| MMP-2 | HS00234422_m1 | Yes | Chr.16: 55513081 - 55540586 | NM_001127891.1 |
| Flt-1 | hs01052961_m1 | Yes | Chr.13: 28874483 - 29069265 | NM_001159920.1 |
| KDR | Hs00911700_m1 | Yes | Chr.4: 55944426 - 55991762 | NM_002253.2 |
| 18S | Hs99999901_s1 | *Both primers and probe map within a single exon* | n/a | X03205.1 |


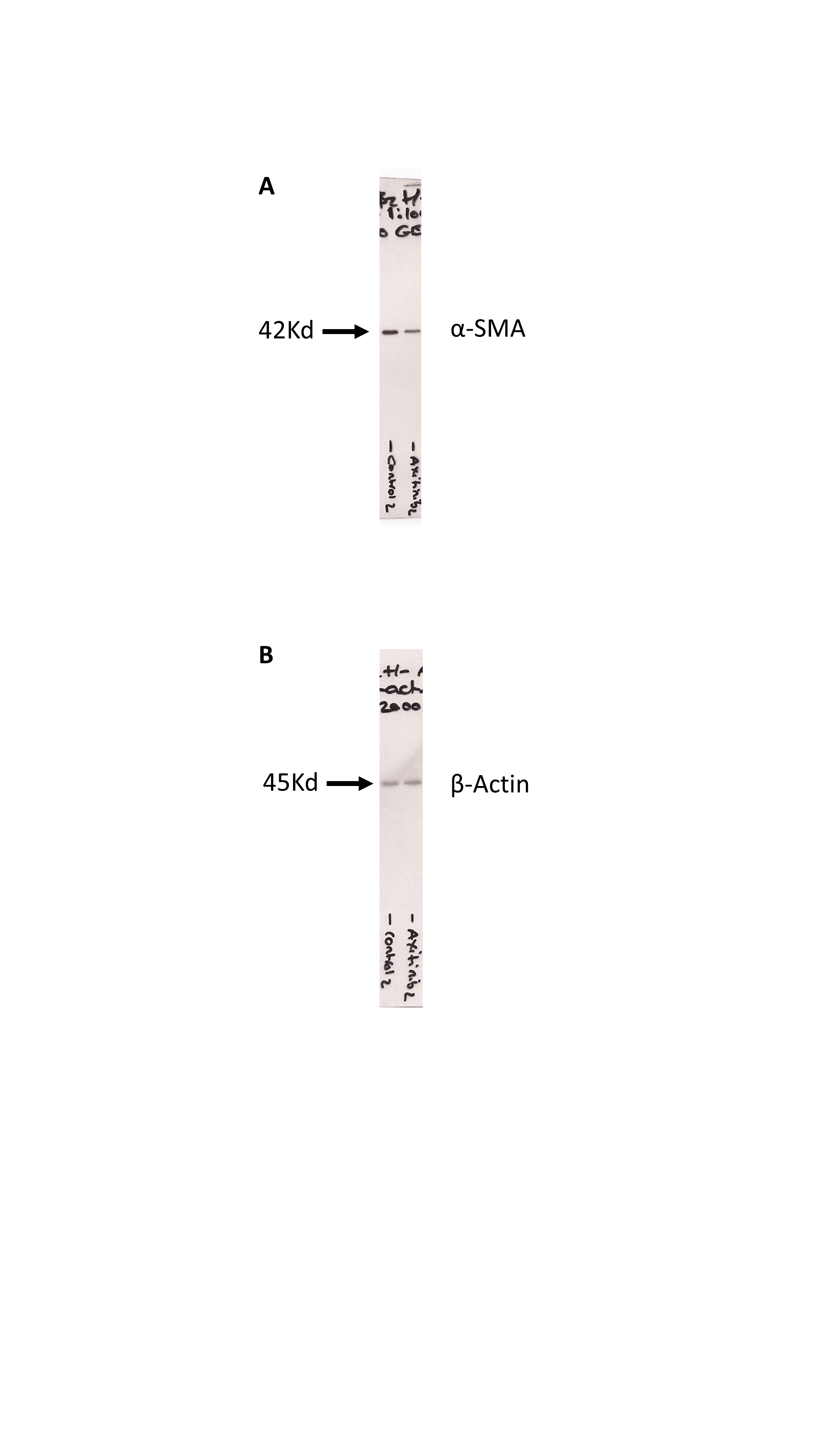


**Supplementary Figure 1.** Full length western blots for (A) αSMA and (B) β-Actin loading control in FHL124 cells maintained in the presence or absence of axitinib (10μM) ) for 48hrs. The antibodies used are well characterised commercial antibodies, which present a single identifiable band, and have been used in this cellular system previously.

**
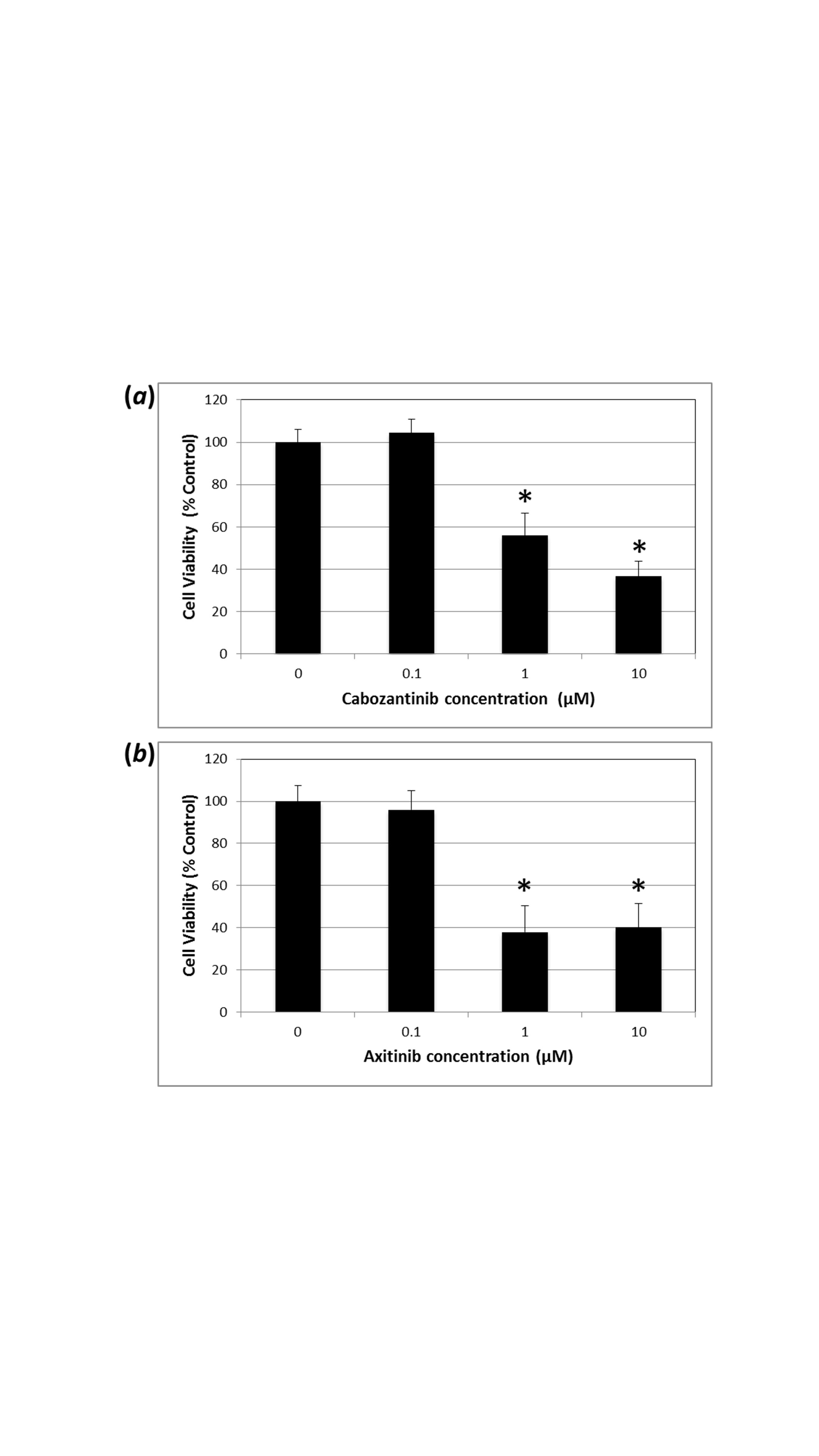
**

**Supplementary Figure 2.** Concentration-dependent effects of VEGFR inhibition on cell viability of FHL124 cells following a 48-hour treatment period, determined using the WST-1 assay. (a) Illustrates VEGFR2 inhibition with Cabozantinib and (b) Pan-VEGFR inhibition with Axitinib. The data is expressed as mean ± SEM (n=3). * Indicates a significant difference between treatment and control (Dunnett’s post hoc analysis; p ≤0.05).


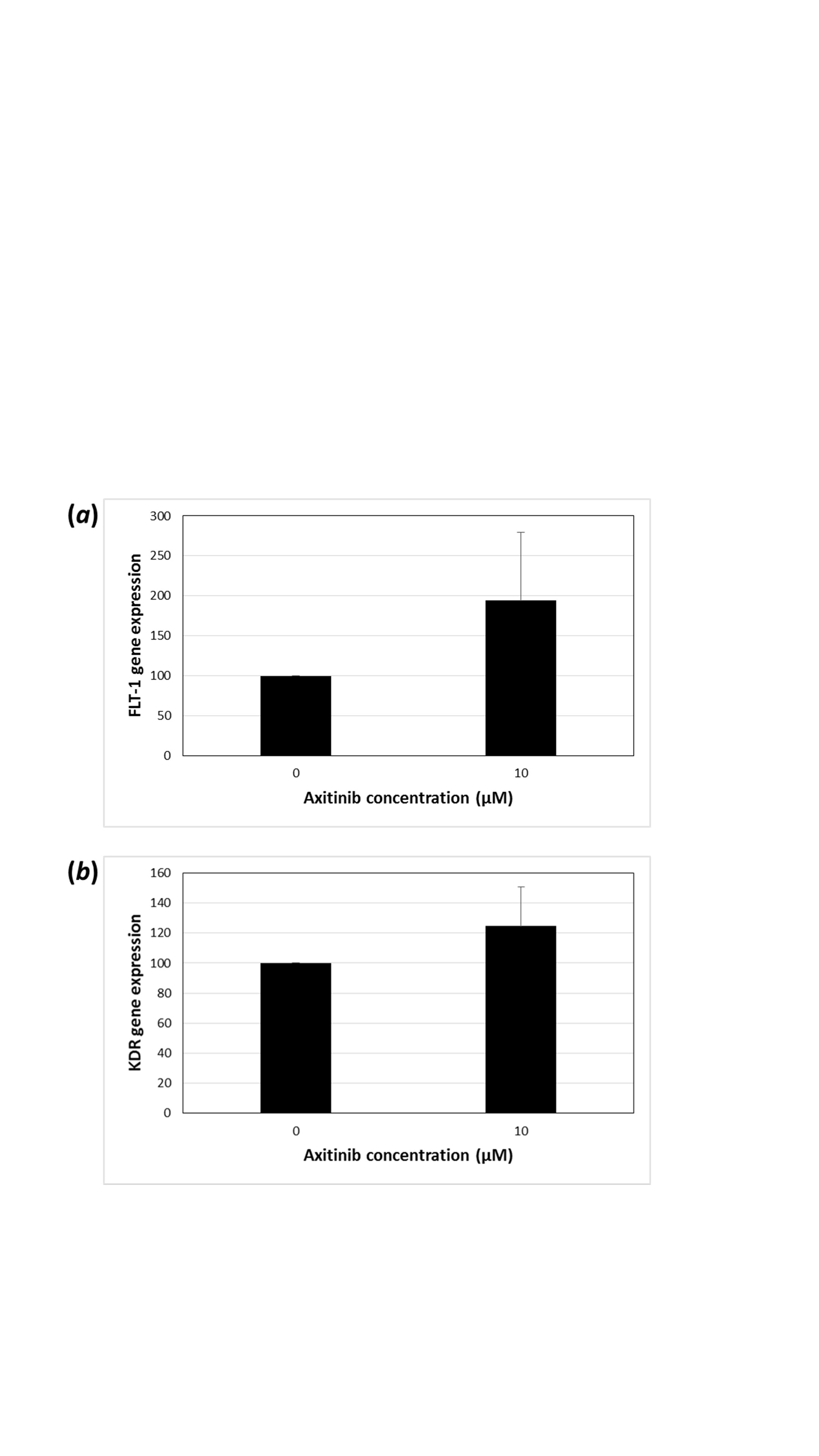


**Supplementary Figure 3.** The influence of VEGF receptor inhibition by Axitinib on (a) VEGFR1/FLT-1 and (b) VEGFR2/KDR gene expression in FHL124 cells, detected using TaqMan real-time PCR. Cells were maintained in experimental conditions for 24 hours and gene expression detected. The data represent mean ± SEM (n=3). No significant difference between control and treated groups was detected (p ≤0.05; Student’s ttest).
